# Independent Effects of Hypertension and Obesity on Left Ventricular Mass and Geometry: Evidence from the Cardiovision 2030 Study

**DOI:** 10.3390/jcm8030370

**Published:** 2019-03-15

**Authors:** Andrea Maugeri, Jana Hruskova, Juraj Jakubik, Martina Barchitta, Oriana Lo Re, Sarka Kunzova, Jose R. Medina-Inojosa, Antonella Agodi, Sergio Sciacca, Manlio Vinciguerra

**Affiliations:** 1 Department of Medical and Surgical Sciences and Advanced Technologies “GF Ingrassia”, University of Catania, Via Santa Sofia 100, 95123 Catania, Italy; andreamaugeri88@gmail.com (A.M.); martina.barchitta@unict.it (M.B.); agodia@unict.it (A.A.); 2 International Clinical Research Center, St’Anne University Hospital, 602 00 Brno, Czech Republic; jana.hruskova@fnusa.cz (J.H.); juraj.jakubik@fnusa.cz (J.J.); oriana.lore@fnusa.cz (O.L.R.); sarka.kunzova@fnusa.cz (S.K.); 3 Department of Cardiovascular Medicine, Division of Preventive Cardiology, Mayo Clinic, Rochester, MN 55905, USA; MedinaInojosa.Jose@mayo.edu; 4 Department for the Treatment and Study of Cardiothoracic Diseases and Cardiothoracic Transplantation, IRCCS-ISMETT, 90127 Palermo, Italy; ssciacca@ismett.edu

**Keywords:** obesity, cardiac hypertrophy, left ventricular remodelling, blood pressure, epidemiology

## Abstract

Obesity and hypertension independently promote pathological left ventricular remodelling (LVR) and left ventricular hypertrophy (LVH), but to what extent they do so when they do not coexist is unclear. We used data from the Cardiovision Brno 2030 study to assess—for the first time in a region where no investigations have been previously carried out—the independent association of obesity and hypertension with LV geometry, and to evaluate the effects of hypertension in normal weight patients and the effects of obesity in normotensive patients. Overall, 433 individuals, aged 25–65 years, with no history of cardiovascular disease and/or antihypertensive treatment, were stratified into four groups according to BMI and hypertension: normal weight non-hypertensive (NWNH), normal weight hypertensive (NWH), overweight/obese non-hypertensive (ONH) and overweight/obese hypertensive (OH). LVR was classified as normal, concentric LVR (cLVR), concentric LVH (cLVH) or eccentric LVH (eLVH). Linear regression analysis demonstrated that body mass index (BMI) and systolic blood pressure (SBP) are the main predictors of LV mass and that they interact: SBP had a stronger effect in overweight/obese (β = 0.195; *p* = 0.033) compared to normal weight patients (β = 0.134; *p* = 0.048). Hypertension increased the odds of cLVR (OR = 1.78; 95%CI = 1.04–3.06; *p* = 0.037) and cLVH (OR = 8.20; 95% CI = 2.35–28.66; *p* = 0.001), independent of age, sex and BMI. Stratified analyses showed that NWH had a greater odd of cLVH (OR = 7.96; 95%CI = 1.70–37.08; *p* = 0.008) and cLVR (OR = 1.62; 95%CI = 1.02–3.34; *p* = 0.047) than NWNH. In the absence of hypertension, obesity was not associated with LVM and abnormal LV geometry, suggesting that it is not per se a determinant of LVR. Thus, antihypertensive therapy still remains the first-line approach against LVH in hypertensive patients, though weight loss interventions might be helpful in those who are obese.

## 1. Introduction

In 1992, Ganau et al. proposed a four-element classification of the left ventricular (LV) geometry of the heart [1]. This classification was based primarily on left ventricular mass index (LVMI) and relative wall thickness (RWT) parameters, and detailed three abnormal left ventricular geometric patterns: Concentric left ventricular remodelling (cLVR), eccentric left ventricular hypertrophy (eLVH) and concentric LVH (cLVH) [1]. Since this description was made, numerous studies have shown that these LV geometric patterns are associated with cardiovascular disease (CVD) and all-cause mortality [1]. LV remodelling (LVR) commonly occurs after events such as myocardial infarction, idiopathic dilated cardiomyopathy, or volume or pressure overload. eLVH is mainly induced by volume overload and is characterized by the addition of new sarcomeres in-series to existing sarcomeres [1]. This effect results in an increase in cavity size while wall thickness remains normal. cLVH, and in the long run cLVR, is mainly induced by pressure overload, such as hypertension [1]. Here, new sarcomeres are added to existing sarcomeres in-parallel. cLVH and cLVR are traditionally considered as conditions associated with severe hemodynamic and structural eLVH abnormalities and represent unfavourable LV adaptation [2]. eLVH, however, may be considered as a physiological adaptation in endurance athletes [2].

Obesity (or BMI >30 Kg/m^2^) is a pandemic that affects 20–30% of the general population and has been consistently and strongly associated with a higher risk of CVD incidence and mortality [3]. In turn, elevated blood pressure (or hypertension) affects 30–45% of the general population and is one of the strongest risk factors for almost all CVDs, including coronary disease, valvular heart diseases, atrial fibrillation and cerebral stroke, irrespective of obesity [4]. Obesity and hypertension are the two major determinants of LVR. Indeed, large-size epidemiological studies have shown that hypertension is the strongest determinant of cLVR and cLVH independent of obesity [5,6]. Conversely, other large population studies revealed that obesity can induce LVR independent of hypertension; but whether obesity-dependent LVR consists of cLVR, cLVH or eLVH, is unclear [7]. As obesity and hypertension often coexist in patients, there is a consensus that their deleterious effects on LVR are additive or even synergistic [8,9]. However, it is unknown to what extent they individually affect LV mass and geometry when they do not coexist.

The identification of risk factors for LVR and easily measurable imaging outcomes could have immediate relevance for public-health strategies aiming to reduce the incidence of CVDs. If LVR precedes preclinical hypertension, the identification of functional abnormalities in obese patients without hypertension might point out a group who could benefit from specific counselling regarding weight loss [7]. By contrast, if hypertension is a predictor of LVR in the absence of obesity, anti-hypertensive drugs should be the frontline treatment to revert pathological LVR, especially in normal weight patients.

Here, we used data from the Cardiovision (“Kardiovize”) 2030 study, a prospective cohort recruited from the urban population of Brno, the second largest city of the Czech Republic, to investigate the complex relationships between CVD risk factors and CVD outcomes in the context of a range of biological, psychosocial, environmental and behavioural factors, [10,11,12,13,14,15]. For the first time in a region where no investigations have been previously carried out, we performed a cross-sectional analysis on Cardiovision participants to confirm whether obesity and hypertension are independently associated with LV mass and geometry. We then conducted a stratified analysis to evaluate the effects of hypertension in normal weight patients and the effects of obesity in normotensive patients.

## 2. Materials and Methods

### 2.1. Study Design

A baseline examination of the Cardiovision Brno 2030 study was completed in 2014, with planned prospective follow-up every 5 years until 2030 [13]. The study protocol was approved by the Ethics Committee of St Anne’s University Hospital, Brno, Czech Republic (reference 2 G/2012) in accordance with the Declaration of Helsinki, and all participants signed an informed consent to participate in the study. The current cross-sectional analysis used data from participants with BMI ≥18.5. All eligible participants completed a physical examination, with assessment of anthropometric, biochemical, and echocardiographic parameters. BMI (calculated as weight in kilograms divided by height in meters squared) was used to classify patients as normal weight (BMI <25 Kg/m^2^) or overweight/obese (BMI ≥25 Kg/m^2^) [16]. Blood pressure was measured using a mercury sphygmomanometer (Baumanometer, W.A. Baum, Co., Inc., Copiague, NY, USA) and used to define hypertension (blood pressure ≥140/90 mmHg). Accordingly, the study population consisted of normal weight normotensive (NWNH), normal weight hypertensive (NWH), overweight/obese normotensive (ONH) and overweight/obese hypertensive (OH) patient groups. To avoid potential confounders, we excluded patients with previous or current CVD and those who received anti-hypertensive treatment.

### 2.2. Physical Examination

A physical examination was performed by trained professionals according to standardized and validated protocols [13]. In brief, height and weight were measured to the nearest 1 cm and 1 kg, respectively, using a medical digital scale with meter (SECA 799; SECA, GmbH and Co. KG, Hamburg, Germany). Waist circumference was measured to the nearest 1 cm by manual tape measurement, and central obesity was defined according to the World Health Organization criteria [17]. Biochemical analyses were performed on 12 h fasting blood samples using a Modular SWA P800 analyzer (Roche, Basel, Switzerland). Total cholesterol, triglycerides, and glucose levels were measured by the enzymatic colorimetric method (Roche Diagnostics GmbH, Mannheim, Germany); high-density lipoprotein (HDL)-cholesterol was measured using the homogeneous method for direct measuring without precipitation (Sekisui Medical, Tokyo, Japan). For triglycerides <4.5 mmol/L, the low-density lipoprotein (LDL)-cholesterol level was calculated according to the Friedewald equation. For triglycerides >4.5 mmol/L, the LDL-cholesterol level was calculated using the homogeneous method for direct measuring (Sekisui Medical, Japan).

### 2.3. Echocardiography

Transthoracic echocardiography was performed with a GE-Vingmed Vivid E9 device (GE Vingmed Ultrasound AS, Horten, Norway) using a 1.5–4.6 MHz sector transducer. Images of the subcostal projections were obtained during quiet breathing or at the end of expiration while the patient was in a left lateral decubitus or supine position. An ECG was recorded and displayed simultaneously, and analysis was performed using EchoPAC PC software version 113. Chamber qualification and Doppler analyses were assessed according to the criteria of the American Society of Echocardiography (ASE) [18,19,20]. According to ASE and the European Association of Cardiovascular Imaging (EACVI) recommendations, LVH was classified depending on gender and weight. LV geometry was classified according to whether LVMI is normal or increased and whether ventricular morphology (RWT) is altered. RWT is variably reported as (posterior wall diameter × 2)/LVd or (IVS + posterior wall)/LVd; this study used the former as septal measurements may be confounded by the presence of a septal bulge. Normal LV geometry is described as RWT <0.42 and LVMI ≤115 g/m^2.7^ for men or ≤95 g/m^2.7^ for women; cLVH is described as RWT >0.42 and LVMI >115 g/m^2.7^ for men or >95 g/m^2.7^ for women; eLVH is described as RWT 0.42 and LVMI >115 g/m^2.7^ for men or >95 g/m^2.7^ for women and cLVR is described as RWT >0.42 and LVMI ≤115 g/m^2.7^ for men or ≤95 g/m^2.7^ for women, respectively [18].

### 2.4. Covariates

Socio-demographic (i.e., age, sex, educational level, marital and employment status) and lifestyle (i.e., smoking status and physical activity) characteristics were collected by face-to-face interviews. Smoking status was categorized as either current (including daily or occasional smokers) or non-smoking (never smoked or not smoked for >12 months). Physical activity was reported as the Metabolic Equivalent of Task (MET-min/week) and was assessed using the long form standardized Czech version of the International Physical Activity Questionnaire (IPAQ-L) [21].

### 2.5. Statistical Analyses 

All statistical analyses were conducted using SPSS software (version 22.0, SPSS, Chicago, IL, USA). Categorical variables were described using frequency (%) and compared using the Chi-square test. The Kolmogorov-Smirnov test was used to test the normality of continuous variables prior to further analyses. Continuous variables underlying a skewed distribution were described using median and interquartile range (IQR) and compared using the Mann-Whitney U test. Spearman’s correlation analysis was performed to determine the correlation between continuous variables and LVMI. A linear regression model was established to identify independent predictors of LVMI, including age, gender and variables that significantly correlated with LVMI in the bivariate correlations. An interaction term was used to test whether BMI and systolic blood pressure had an interactive effect on LVMI. Logistic regression models were used to evaluate (i) the associations of systolic blood pressure or hypertension with abnormal LV geometry patterns, after adjustment for age, sex and BMI; (ii) the associations of BMI or obesity with abnormal LV geometry patterns, after adjustment for age, sex and systolic blood pressure. Linear and logistic regression models were either performed on the whole cohort or stratified by BMI categories. All statistical tests were two-sided, and *p* values <0.05 were considered statistically significant.

## 3. Results

### 3.1. Study Population

The Cardiovision Brno 2030 cohort included 433 participants (50.1% males), aged 25 to 65 years, who satisfied the selection criteria and could be included in the current analysis. According to BMI and hypertension, we classified 47.1% participants as normal weight normotensive (NWNH), 8.5% as normal weight hypertensive (NWH), 30.5% as overweight/obese normotensive (ONH) and 13.9% as overweight/obese hypertensive (OH) (Table 1). Beyond blood pressure, NWH patients were older (*p* = 0.028) and exhibited a higher BMI (*p* = 0.034), waist circumference (*p* = 0.012) and fasting glucose (*p* = 0.014) than NWNH patients. Compared with NWNH patients, ONH were more likely to be men (*p* <0.001) and were less educated (*p* = 0.002), OH were older (*p* <0.001), and both exhibited higher anthropometric and biochemical measures (*p*-values <0.001). Except for hypertension and age (*p* = 0.004), we found no significant differences between ONH and OH patients.

### 3.2. BMI and Systolic Blood Pressure Are Strong Predictors of Left Ventricular Mass Index

We first aimed to determine the predictors of LVMI. To do so, we performed univariate analysis, which showed significant correlations between LVMI and age, BMI, waist circumference, SBP, DBP, fasting glucose, triglycerides and HDL-cholesterol. In the sex-adjusted linear regression model, only BMI and systolic blood pressure maintained independent associations with LVMI (Table 2). In addition, we observed a significant interaction between BMI and systolic blood pressure on LVMI (*p* = 0.002), with a stronger effect of systolic blood pressure in overweight/obese patients (β = 0.195; *p* = 0.033) compared to normal weight patients (β = 0.134; *p* = 0.048). In contrast, BMI seemed to be associated with LVMI in hypertensives (β = 0.112; *p* = 0.046) but not in non-hypertensive subjects (β = 0.089; *p* = 0.128).

### 3.3. ECG Parameters According to BMI and Hypertension

We next detailed the ECG parameters of all patients according to BMI and hypertension (Table 3) in order to understand their difference according to the established categories of ONH, OH, NWNH and NWH. In brief, we found that RWT, LVM and LVMI were all significantly higher in NWH, ONH and OH compared to NWNH patients (Table 3). These data suggest that hypertension and obesity, alone or in combination, affect ECG parameters.

### 3.4. Left Ventricular Geometry According to BMI and Hypertension

**Figure 1:**
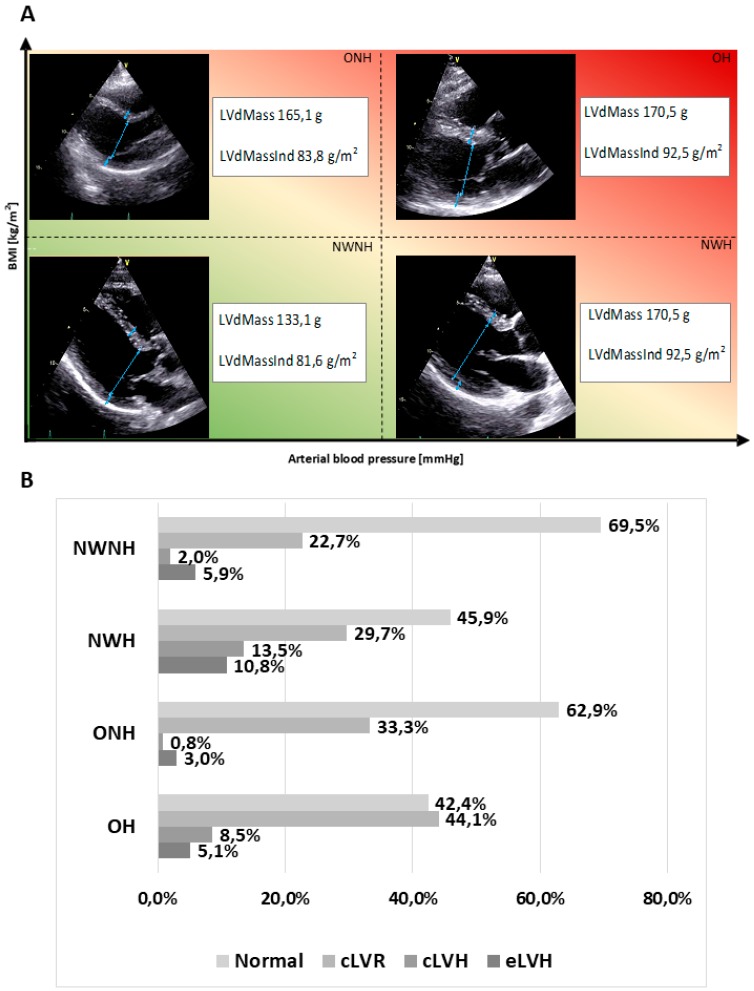
Representative echo images (**A**) and quantitative assessment (**B**) of the variations in LV geometry parameters based on arterial blood pressure and BMI. Visual emphasis on various LV geometry (**A**) within four chosen categories, through schematic drawings based on real echo images.

We next assessed the relative variations in LV geometry according to arterial blood pressure and BMI (Figure 1), in order to understand their structural impact on the heart. Among the abnormal LV geometry patterns, concentric remodelling was the most prevalent pattern in all groups, ranging from 22.7% in NWNH to 44.1% in OH. NWH showed a significantly higher prevalence of cLVH (13.5%) compared to NWNH (2.0%; *p* <0.001). Similarly, we observed a significantly higher prevalence of cLVR and cLVH in OH compared to NWNH patients (44.1% vs. 22.7%; *p* <0.001 and 8.5% vs. 2.0%; *p* = 0.002, respectively). By contrast, we found no significant differences in the prevalence of abnormal LV geometry patterns between ONH and NWNH patients. These data indicate that BMI is not per se a determinant of pathological remodelling.

### 3.5. Association of Hypertension and Obesity with Left Ventricular Geometry

We next performed logistic regression analyses to determine the association between blood pressure and LV geometry. Overall, we found that a one-unit increase in systolic blood pressure was significantly associated with cLVR (OR = 1.023, 95%CI = 1.005–1.041; *p* = 0.012), cLVH (OR = 1.054, 95%CI = 1.013–1.097; *p* = 0.010) and eLVH (OR = 1.033, 95%CI = 1.001–1.066; *p* = 0.042), independent of age, sex and BMI. Consistently, hypertension increased the odds of cLVR (OR = 1.78; 95%CI = 1.04–3.06; *p* = 0.037) and cLVH (OR = 8.20; 95%CI = 2.35–28.66; *p* = 0.001), but not of eLVH (OR = 2.40; 95%CI = 0.88–6.59; *p* = 0.089). In a similar model, neither BMI nor obesity were associated with abnormal LV geometry patterns, independent of age, sex and systolic blood pressure. In line with this evidence, stratified analyses showed that NWH patients had greater odds of cLVR (OR = 1.62; 95%CI = 1.02–3.34; *p* = 0.047) and cLVH (OR = 7.96; 95%CI = 1.70–37.08; *p* = 0.008) than NWNH patients. Similarly, OH patients had greater odds of cLVR (OR = 2.89; 95%CI = 1.43–5.83; *p* = 0.003) and cLVH (OR = 5.13; 95%CI = 1.09–24.14; *p* = 0.039) than NWNH patients, but also greater odds of cLVH than ONH (OR = 13.05; 95%CI = 1.32–128.45; *p* = 0.028). By contrast, we found no association between ONH and abnormal LV geometry patterns when compared with NWNH patients. In sum, these data confirm a prevalent role for hypertension over obesity in inducing pathological LVR.

## 4. Discussion

In line with the current consensus [8,9], our study has confirmed—for the first time in a region where no investigations have been previously carried out—that obesity and hypertension are the major determinants of LVR and LVH. Among European countries, Czech Republic figures at the top for incidence of obesity, and its population performs very poorly in metabolic and cardiovascular health [10,11,12]. In this cohort, we observed that LVM, LVMI and RWT were significantly higher in NWH and OH compared to NWNH patients. These results suggest that hypertension, alone or in combination with obesity, negatively affect ECG parameters. Accordingly, we also found a significant interaction between obesity and hypertension that enhanced the deleterious effect of high blood pressure in overweight and obese patients. Indeed, obese individuals often develop systemic hypertension and pressure overload, which exert an exponential effect on the prevalence of LV hypertrophy [22]. In line with this evidence, previous studies reported that LVH ranged between ~15% in ONH and over 75% in OH [23]. Either systolic blood pressure or hypertension also significantly increased the odds of cLVR and cLVH, independent of age, sex and BMI. This obesity-independent effect of hypertension on concentric LV geometry has been previously recognized by several epidemiological studies [5,6]. Our stratified analysis has delved further to determine that hypertension is associated with increased odds of cLVR and cLVH in normal weight patients. Similarly, we found that OH patients had greater odds of cLVR and cLVH compared with NWNH patients. This last finding is consistent with previous studies [8,24] demonstrating that the coexistence of obesity and hypertension substantially increases the prevalence of cLVR and cLVH.

With respect to obesity, previous studies have suggested that BMI was positively correlated with LVM after adjusting for blood pressure [25], and that LVH, especially eLVH, was more prevalent in normotensive obese patients than in their lean counterpart [26]. However, further studies failed to provide unequivocal findings [27], and hence it still remains unknow whether obesity affects LV mass and geometry in the absence of hypertension. In reference to this question, we found that ONH patients exhibited increased ECG parameters, including LVM and LVMI, compared to NWNH patients. It is important to highlight, however, that ONH patients still had significantly higher blood pressures than NWNH, but not in the hypertensive range. Thus, the pressure load in obese patients was consistently higher than in normal-weight patients, even in the presence of blood pressure levels within the normal range. These findings are in line with Avelar et al., who speculated that even mild increases in blood pressure within the normal range may have deleterious effects on LV mass in obese subjects [8]. Despite this evidence, our stratified analysis demonstrated that LVMI increased with increasing BMI only in hypertensive patients. Overall, our results add to the current debate about whether obesity-dependent LV remodelling consists of concentric or eccentric geometry. The traditional paradigm considers that a predominant volume load results in eLVH [28], which is characterized by a normal mass/volume ratio and relative wall thickness. Previous data obtained from obese normotensive patients showed that eLVH is more common than cLVH [29], but more recent analyses have proposed that concentric rather than eLVH might be the predominant LV pattern in obese patients [29]. A plausible explanation for this controversy is that studies showing a predominance of cLVR or cLVH were unable to adjust for or exclude patients with hypertension [7]. By exploiting stratified analyses and excluding patients treated for hypertension, we instead found no significant difference in the prevalence of abnormal LV geometry between obese and normal weight patients in the absence of hypertension. eLVH was, however, more common than cLVH in ONH patients. By contrast, OH patients had higher odds of cLVH than ONH patients. These data suggest that BMI is not per se a determinant of pathological remodelling.

The low prevalence of LVH, especially in ONH patients, could be a weakness of our study. Moreover, the cross-sectional design does not allow us to yet understand the mechanisms that cause LVR and LVH in obesity and hypertension. Furthermore, large and prospective studies are now needed to understand how obesity and hypertension contribute both independently and additively to pathological LVR. Prevalence of LVH is higher in the elderly, particularly since hypertension and obesity increase with age [30,31]. Moreover, the effect of hypertension on LVM may differ by gender [32,33], thus that hypertensive women are more likely to develop cLVH than their male counterparts [33]. For these reasons, we performed multivariate analyses adjusting for age, gender, and those variables that were significantly associated with LVM in bivariate analysis. Despite this, we cannot rule out the possibility of bias from residual confounders that might affect the relationship between obesity, hypertension and LVR. Finally, the best approach to define LVH for risk prediction and reduction is currently a matter of debate, namely whether LVH defined using height is preferable to LVH defined using LV mass indexed to body surface area, which is greater in obese subjects compared to normal weight subjects [9,34]. In the case of a high prevalence of obesity, the population risk attributable to LVH identified by normalization of LV mass to height^2.7^ is substantially greater than by normalization to body surface area [35]. However, LVH has prognostic relevance in all subjects irrespective of obesity status. As previously proposed [36], the best method of normalization could be free fat mass, but it is not routinely measured. Height, which is the main factor to contribute to the magnitude of free fat deposition, could be a surrogate of free fat mass. In the absence of general consensus, we adopted the method based on normalization of LVM based on height.

## 5. Conclusions

In conclusion, our study supports the notion that hypertension is the major determinant of LVM and pathological LVR. Thus, blood pressure control with long-term antihypertensive therapies (e.g., angiotensin-converting enzyme inhibitors, angiotensin II receptor blockers, and calcium channel blockers) still remains the first-line approach against LVH in hypertensive patients [37,38]. In line with current evidence [27], our findings also confirm a joint effect of hypertension and obesity on LVM and LVR, with higher prevalence of cLVR and cLVH in obese hypertensive patients than in their lean normotensive counterparts. Since weight reduction might decrease LVM in obese hypertensive patients [7], lifestyle and dietary interventions might support blood pressure control but also the prevention of LVH. In the absence of hypertension, however, no significant difference in LV geometry were evident between obese and normal weight patients, and hence obesity was not per se a determinant of pathological remodelling. Further research is encouraged to elucidate to what extent obesity affects pathological LVR when it does not coexist with hypertension.

## Figures and Tables

**Table 1:** Characteristics of the study population.

Characteristics	NWNH (*n* = 204)	NWH (*n* = 37)	*p*-Value	ONH (*n* = 132)	*p*-Value	OH (*n* = 60)	*p*-Value
Age, years	42.0 (16.0)	49.0 (19.0)	0.028	44.5 (16.3)	0.244	52.0 (17.5)	<0.001
Sex (% male)	38.2%	48.6%	0.234	64.4%	<0.001	60.0%	0.003
Education level (% low)	7.8%	5.4%	0.138	11.5%	0.002	11.7%	0.567
Marital status (% living alone)	41.2%	37.8%	0.704	35.1%	0.267	26.7%	0.042
Employment (% unemployed)	12.5%	13.5%	0.895	8.5%	0.056	13.6%	0.266
Smoking (% current smokers)	21.6%	35.1%	0.310	15.9%	0.238	11.7%	0.128
Physical activity, MET-min/week	2983 (3363)	34485 (5731)	0.523	3555 (5593)	0.355	3297 (3736)	0.637
Weight, Kg	66.0 (14.0)	68.0 (10.0)	0.135	88.0 (14.0)	<0.001	88.0 (21.0)	<0.001
BMI, Kg/m^2^	21.9 (2.8)	23.0 (2.3)	0.034	27.4 (2.8)	<0.001	27.9 (4.2)	<0.001
Waist circumference, cm	78.0 (11.0)	82.9 (12.0)	0.012	95.0 (10.0)	<0.001	99.0 (17.0)	<0.001
Fat mass (%)	20.3 (10.8)	21.8 (9.3)	0.285	25.0 (13.8)	<0.001	28.0 (13.5)	<0.001
Central Obesity (%)	0.0%	0.0%	-	34.8%	<0.001	48.3%	<0.001
Systolic Blood Pressure, mmHg	111.0 (13.0)	131.0 (21.0)	<0.001	115.0 (10.5)	<0.001	138.3 (18.4)	<0.001
Diastolic Blood Pressure, mmHg	74.0 (11.0)	87.5 (11.5)	<0.001	77.5 (10.0)	<0.001	91.0 (7.4)	<0.001
Fasting Glucose, mmol/L	4.7 (0.7)	4.8 (056)	0.014	4.9 (0.6)	<0.001	4.9 (0.6)	<0.001
Triglycerides, mmol/L	0.85 (0.40)	0.86 (0.70)	0.141	1.21 (1.0)	<0.001	1.24 (0.9)	<0.001
Total Cholesterol, mmol/L	4.9 (1.4)	5.0 (1.5)	0.457	5.4 (1.6)	<0.001	5.2 (1.3)	0.004
HDL Cholesterol, mmol/L	1.6 (0.5)	1.6 (0.5)	0.946	1.3 (0.5)	<0.001	1.4 (0.5)	<0.001
LDL Cholesterol, mmol/L	2.9 (1.1)	2.8 (1.2)	0.754	3.4 (1.4)	<0.001	3.3 (1.2)	<0.001

Abbreviations: Normal weight normotensive, NWNH; normal weight hypertensive, NWH; overweight/obese normotensive, ONH; overweight/obese hypertensive, OH; metabolic equivalent task, MET.0.

**Table 2:** Univariate and multivariate predictors of left ventricular (LV) mass index.

Characteristics	Bivariate Correlation		Linear Regression	
	Spearman Correlation Coefficient	*p*-Value	Standardized Coefficient (β)	*p*-Value
Age, years	0.097	0.043	0.040	0.353
BMI, Kg/m^2^	0.207	<0.001	0.171	0.037
Waist circumference, cm	0.292	<0.001	0.167	0.087
Systolic Blood Pressure, mmHg	0.253	<0.001	0.153	0.023
Diastolic Blood Pressure, mmHg	0.242	<0.001	−0.027	0.676
Fasting Glucose, mmol/L	0.120	0.013	−0.006	0.883
Triglycerides, mmol/L	0.128	0.008	−0.026	0.533
HDL Cholesterol, mmol/L	−0.246	<0.001	−0.041	0.344
LDL Cholesterol, mmol/L	−0.004	0.928	-	-

**Table 3:** Echocardiographic parameters of the study population.

Characteristics	NWNH (*n* = 204)	NWH (*n* = 37)	*p*-Value	ONH (*n* = 132)	*p*-Value	OH (*n* = 60)	*p*-Value
LVM	133.1 (51.9)	145.0 (55.8)	0.015	165.1 (44.1)	<0.001	170.5 (59.9)	<0.001
LVMIe	81.6 (24.3)	82.7 (31.3)	0.048	83.8 (19.6)	0.017	92.5 (22.5)	0.001
RWT	0.37 (0.08)	0.42 (0.08)	0.002	0.40 (0.09)	0.012	0.43 (0.09)	0.003

Abbreviations: Normal weight normotensive, NWNH; normal weight hypertensive, NWH; overweight/obese normotensive, ONH; overweight/obese hypertensive, OH; LVMI, left ventricular mass index, LVMI; relative wall thickness, RWT.
